# Effectiveness of Low-Level Laser Therapy Associated with Strength Training in Knee Osteoarthritis: Protocol for a Randomized Placebo-Controlled Trial

**DOI:** 10.3390/mps4010019

**Published:** 2021-03-01

**Authors:** Martin Bjørn Stausholm, Ingvill Fjell Naterstad, Christian Couppé, Kjartan Vibe Fersum, Ernesto Cesar Pinto Leal-Junior, Rodrigo Álvaro Brandão Lopes-Martins, Jan Magnus Bjordal, Jon Joensen

**Affiliations:** 1 Department of Global Public Health and Primary Care, University of Bergen, 5009 Bergen, Norway; Ingvill.Naterstad@uib.no (I.F.N.); Kjartan.Fersum@uib.no (K.V.F.); Ernesto.Junior@uib.no (E.C.P.L.-J.); Jan.Bjordal@uib.no (J.M.B.); Jon.Joensen@uib.no (J.J.); 2 Physical and Occupational Therapy Research Unit, Bispebjerg and Frederiksberg University Hospital, 2400 Copenhagen, Denmark; Christian.Couppe@regionh.dk; 3 Laboratory of Phototherapy and Innovative Technologies in Health, Post-Graduate Program in Rehabilitation Sciences, Nove de Julho University, São Paulo 01504-001, Brazil; 4 Physical de Pesquisa & Desenvolvimento, Universidade do Vale do Paraíba, São José dos Campos 12244-390, Brazil; Rodrigo@univap.br

**Keywords:** inflammation, knee osteoarthritis, low-level laser therapy, LLLT, strength training

## Abstract

Physical activity and low-level laser therapy (LLLT) can reduce knee osteoarthritis (KOA) inflammation. We are conducting a randomized placebo-controlled trial to investigate the long-term effectiveness of LLLT combined with strength training (ST) in persons with KOA, since it, to our knowledge, has not been investigated before. Fifty participants were enrolled. LLLT and ST was performed 3 times per week over 3 and 8 weeks, respectively. In the LLLT group, 3 Joules of 904 nm wavelength laser was applied to 15 spots per knee (45 Joules/knee/session). The primary outcomes are pain during movement, at night and at rest (Visual Analogue Scale) and global pain (Knee injury and Osteoarthritis Outcome Score, KOOS) pain subscale. The secondary outcomes are KOOS disability and quality-of-life, analgesic usage, global health change, knee active range of motion, 30 s chair stand, maximum painless isometric knee extension strength, knee pain pressure threshold and real-time ultrasonography-assessed suprapatellar effusion, meniscal neovascularization and femur cartilage thickness. All the outcomes are assessed 0, 3, 8, 26 and 52 weeks post-randomization, except for global health change, which is only evaluated at completed ST. This study features the blinding of participants, assessors and therapists, and will improve our understanding of what occurs with the local pathophysiology, tissue morphology and clinical status of persons with KOA up to a year after the initiation of ST and a higher 904 nm LLLT dose than in any published trial on this topic.

## 1. Introduction

Knee osteoarthritis (KOA) is a common joint disease in the middle-aged and elderly population [1]. It is a complex inflammatory disorder involving pathological changes to the entire knee joint and is associated with, for example, muscle weakness, pain, disability and reduced quality-of-life (QoL) [1]. Inflammatory mediators, including interleukins, can activate the metalloproteinases of chondrocytes, which promotes cartilage deterioration [2]. In KOA, a greater expression of inflammatory markers is associated with more intense pain and more rapid disease progression [1,2]. This advocates the use of anti-inflammatory interventions in osteoarthritis [1,2].

Low-level laser therapy (LLLT) is a non-pharmacological intervention capable of reducing osteoarthritis inflammation in vivo [3,4,5,6]. This could be the reason why in vivo results of a systematic review show that laser therapy of relatively low intensity (<1000 mW/cm^2^) may have a positive effect on osteoarthritis cartilage [7]. Nevertheless, LLLT is not recommended in major treatment guidelines for KOA, such as those by the European League Against Rheumatism (EULAR) and the Osteoarthritis Research Society International (OARSI) [8,9]. We recently published a systematic review with a meta-analysis of placebo-controlled trials showing that LLLT can reduce KOA pain and disability. The trials included in the review were subgrouped by adherence and non-adherence with the World Association for Laser Therapy (WALT) treatment recommendations for LLLT dose per treatment spot [10,11]. The recommended doses provided a significant pain reduction greater than 20 mm on the 0–100 mm Visual Analogue Scale (VAS) versus the placebo from therapy week 4–8 through follow-ups 6–8 weeks after the end of therapy, whereas the non-recommended doses provided no or little pain reduction [12]. However, it is unclear whether LLLT has long-term positive effects in KOA, as it has only been investigated in three of the included studies [12,13,14,15].

Previous LLLT KOA reviews have led to conflicting results, however, they lack a valid dose–response investigation [16,17]. Exercise therapy is widely recommended for persons with KOA [8,9] and can reduce KOA inflammation, although on a smaller scale than NSAID and LLLT [3,4]. A recent systematic review demonstrated that in KOA, exercise interventions following the American College of Sports Medicine (ACSM) definition of strength training (ST) is superior in increasing leg strength compared to other exercise programs [18]. The ACSM recommends that persons with KOA perform at least two ST sessions per week, comprised of 2–4 sets with 8–12 repetitions maximum (RM) to muscle exhaustion [19].

We searched systematically for reports of trials on the topic [12] and found that the effectiveness of LLLT associated with an ACSM ST program in KOA had only been investigated in a few placebo-controlled randomized clinical trials (RCT) and that they did not include long-term assessments [20,21]. Therefore, we set out to investigate the short- and long-term effectiveness of LLLT associated with an ACSM ST program in persons with KOA in a placebo-controlled RCT. Pain was selected as the primary outcome as this is the dominating KOA complaint [22].

## 2. Materials and Methods

### 2.1. Methods and Design

This RCT protocol has been approved by the Research Ethical Committee North (reference 2017/2417), is registered on the website ClinicalTrials.gov (reference NCT03750279) and reported in adherence to the Standard Protocol Items: Recommendations for Interventional Trials guidelines. The intervention is complete and follow-up assessments are ongoing.

### 2.2. Participants

Eligible subjects were recruited from the Bergen municipality (Norway) through written and verbal advertisement.

The inclusion criteria were women and men aged ≥50 years with pain during movement corresponding to ≥40 mm on the Visual Analogue Scale (VAS), knee pain in the last ≥3 months and KOA according to the American College of Rheumatology clinical criteria [23]. The exclusion criteria were knee alloplastic, total meniscectomy, intra-articular steroid injection and/or oral steroid treatment within the last 6 months, cancer, rheumatoid arthritis, severe cognitive deficit, neurological deficits in the lower limb, the inability to speak and understand both English and Nordic and the absence of signed informed consent.

## 3. Procedure

### 3.1. Randomization

Eligible subjects willing to participate in the trial were randomly divided in two parallel groups, one group with ST and LLLT and one group with ST and placebo LLLT. This was carried out after the baseline assessment by drawing of concealed opaque envelopes, each containing a red or green label (group code). The envelopes were prepared by an assistant who will not otherwise be involved in the study. The allocation ratio was 1:1.

### 3.2. Strength Training

All the participants were encouraged to exercise 3 times per week for 8 weeks. The exercises were performed under supervision by a physiotherapist in a clinic 3 times per week in the first 3 weeks and once per week in the subsequent 5 weeks (15 supervised and 9 unsupervised ST sessions). The program does not involve special equipment, except for an elastic band, which is distributed to the participants. This makes the program feasible in a home setting. Each session consisted of 5 min warm up with light weight bearing exercises for the lower limb (sideways walk, stepping and two-legged knee bends), followed by ST level 1 or 2. The participants completed ST level 1 in the first session and were subsequently allowed to interchange between the two levels, if advised by the physiotherapist who took symptom development into account.

1. ST level 1: Pelvic lifts (2 × 15 RM), one-legged knee bends with maximum 60° flexion (2 × 10 RM per leg) and hip abductions with elastic band (2 × 10 RM per leg).
2. ST level 2: Pelvic lifts (3 × 15 RM), one-legged knee bends with maximum 60° flexion (3 × 10 RM per leg), hip abductions with elastic band (2 × 10 RM per leg), sideways slide lunges (2 × 10 RM per leg) and backward slide lunges (2 × 10 RM per leg).

### 3.3. Laser Therapy and Blinding

**Figure 1:**
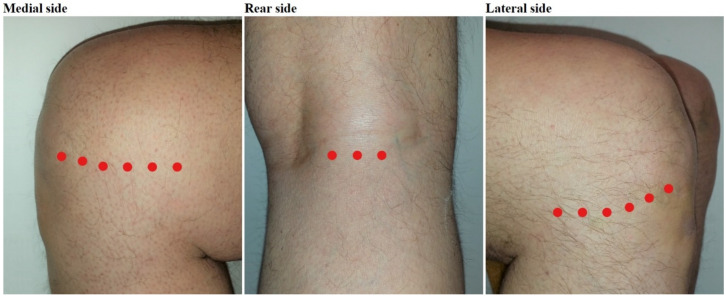
Treatment spots.

The participants in the intervention group received LLLT 3 times per week in the first 3 weeks with an Irradia GaAs laser class 3B device in accordance with the WALT treatment guidelines, in terms of dose per treatment spot: 6 spots in the medial knee joint line, 6 spots in the lateral knee joint line and 3 spots in the popliteal fossa were irradiated with pulsed 904 nm wavelength laser for 50 s with a mean intensity of 60 mW, resulting in 3 Joules per spot, that is, 45 Joules per knee per session (Figure 1). The wavelength is invisible for the naked eye and the low output power does not produce noticeable heat [24]. The participants in the control group were treated with a sham laser device with identical appearance, using the same procedure, but without output power (0 mW) due to a cut wire. The laser devices were provided with a random color code by an assistant, who will not otherwise be involved in the study. These procedures ensure the blinding of the participants and research personnel, including the assessors (M.B.S. and J.J.) and therapists. The participants were accompanied by a maximum of one research personnel at a time. The code for placebo and real LLLT will be revealed after the statistical analyses are complete.

### 3.4. Concomitant Interventions

The participants were asked to avoid receiving additional physiotherapy in the first 8 weeks of the study (intervention period). Furthermore, the participants are not allowed to receive laser therapy in the follow-up period.

The types of other knee interventions made use of by participants during the study are also registered.

### 3.5. Outcomes

The primary outcomes are pain during movement, at night and at rest registered with VAS and global pain measured using the Knee injury and Osteoarthritis Outcome Score (KOOS) pain subscale. The secondary outcomes are KOOS disability and quality-of-life (QoL), analgesic usage, global health change, knee active range of motion (AROM), number of chair stands in 30 s, maximum pain-free isometric knee extension strength, joint line and tibia condyle pain pressure threshold (PPT) and real-time ultrasonography (RTU)-assessed suprapatellar effusion, meniscal neovascularization (Doppler) and femur cartilage thickness.

All the outcomes are assessed 0, 3, 8, 26 and 52 weeks after randomization, except for global health change, which is only evaluated at completed ST (week 8) (Table 1). The sequence of the assessment was typically as follows: Firstly, the participants filled out questionnaires, then ultrasonography was performed and lastly the physical examination was carried out.

#### 3.5.1. VAS (Pain)

The VAS displays “no pain” at one end and “worst imaginable pain” at the other end of the scale and has proven to be more reliable than the Numeric Rating Scale in the assessment of KOA patients [25]. We opted to utilize the VAS digitally rather than in physical format, as it is more convenient and produces the same results [26].

#### 3.5.2. KOOS (Pain, Physical Function, QoL and Other Symptoms)

The KOOS questionnaire is a valid and reliable disease-specific tool based on Likert scales and is comprised of five subscales (global pain, physical function in daily living, physical function in sports and recreational activities, QoL and other symptoms), and the results are converted to 0–100 percentages, where a higher score is better [27].

#### 3.5.3. Global Health Change

Global health change is scored by asking the participants whether they experience no symptoms, a large improvement, some improvement, no change, some worsening, a large worsening or worse symptoms than ever.

#### 3.5.4. Analgesics

Analgesics usage (NSAIDs, paracetamol, etc.) in the last week due to knee pain is scored dichotomously.

#### 3.5.5. AROM

Knee AROM is measured with the participant in supine position using a 2 × 30 cm goniometer, since shorter versions are less reliable [28].

#### 3.5.6. 30 Second Chair Stand Test

The 30 second chair stand test is performed to assess physical function in people with knee osteoarthritis, since this is recommended by the OARSI [29], and the last attempts will count if the participants are more than half-way up.

#### 3.5.7. Maximum Pain-Free Isometric Knee Extension Strength

Maximum pain-free isometric knee extension strength is measured using a hand-held dynamometer (JTech Commander, Midvale, UT, USA) with the participant in a sitting position and the knee in a 90° angle. The dynamometer display is not visible during the measurements to blind the assessor and participant for the levels of force. The dynamometer can measure up to 112.54 N.

#### 3.5.8. PPT

The PPT of the most tender spot on the knee joint line identified by palpation and 1.5 cm distally from this spot (on the tibia condyle) is measured using an algometer (Wagner FPX 25, Greenwich, CT, USA) with a 1 cm^2^ rubber tip. The algometer display is not visible during the measurements to blind the assessor and participant for the levels of force.

#### 3.5.9. RTU

A RTU device (Mindray Diagnostic Ultrasound System M7, Shenzhen, China) is utilized to assess suprapatellar effusion with 30° knee flexion, meniscal neovascularization with 30° knee flexion and femur cartilage thickness with orthogonal probe insonation and maximum knee flexion. The effusion will be scored as its maximum height, the meniscal neovascularization will be quantified as the Doppler pixel area and femur cartilage thickness will be measured at the medial condyle, lateral condyle and patellofemoral grove. We will correct for cartilage thickness as recommended by Torp-Pedersen et al. [30].

### 3.6. Statistial Analysis

Outcome data will be analyzed with the intention-to-treat approach. The distribution of outcome data will be assessed for normality using histograms. Paired and unpaired parametric continuous data will be analyzed with a two-way and one-way analysis of variance (ANOVA), respectively. Paired and unpaired non-parametric continuous outcome data will be analyzed with the Wilcoxon and Mann–Whitney U test, respectively.

### 3.7. Sample Size

We expect a between-group difference in pain during movement of 20 mm VAS [12] and assume that the related standard deviations will be 14.85 mm in the intervention group and 13.93 mm in the control group at completed therapy [31,32,33]. We expect a between-group difference in pain at rest of 15 mm VAS [12] and assume that the related standard deviation will be 15.43 mm in the intervention group and 12.87 mm in the control group at completed therapy [31,32,33]. If correct, an 80% chance to detect a significant difference in pain during movement and pain at rest will require a total of 20 and 32 participants, respectively. A total of 50 subjects will be enrolled to increase the external validity and account for possible dropouts. No power calculation was made for pain at night and global pain (KOOS) as these have not been used as outcomes in a similar study.

## 4. Discussion

This study will improve our understanding of what occurs with the local pathophysiology, tissue morphology and clinical status of persons with KOA up to a year after the initiation SET associated with LLLT.

Our study features a random and concealed group allocation, blinding of participants, assessors and therapists and intention-to-treat analysis, methods of high standard. Although previous studies of the current topic generally appear to have been conducted with low risk of bias, therapist blinding has often lacked [21,33,34,35,36,37]. Our study is not without limitations. Only one laser dose is tested out and other relevant inflammatory markers than meniscal neovascularisation (Doppler) and suprapatellar effusion are not evaluated, such as prostaglandin E2 and interleukin 1 and 6. Furthermore, the long-term results may be impacted by the use of contaminant interventions.

## Figures and Tables

**Table 1:** Timetable of outcome measures.

	Week 0	Week 3	Week 8	Week 26	Week 52
Pain during movement (VAS)	√	√	√	√	√
Pain at night (VAS)	√	√	√	√	√
Pain at rest (VAS)	√	√	√	√	√
Global pain (KOOS)	√	√	√	√	√
Disability in ADL (KOOS)	√	√	√	√	√
Disability in sports/recreation (KOOS)	√	√	√	√	√
Global health change			√		
Analgesic usage	√	√	√	√	√
Knee active range of motion	√	√	√	√	√
30 s chair stand	√	√	√	√	√
Pain-free isometric knee extension strength	√	√	√	√	√
Joint line PPT	√	√	√	√	√
Tibia condyle PPT	√	√	√	√	√
Suprapatellar effusion (RTU)	√	√	√	√	√
Meniscal neovascularization (RTU)	√	√	√	√	√
Femur cartilage thickness (RTU)	√	√	√	√	√

Abbrevations: KOOS: Knee injury and Osteoarthritis Outcome Score; PPT: pain pressure threshold; RTU: real-time ultrasonography; VAS: Visual Analogue Scale.

## Data Availability

Not applicable.
